# Characteristics of Biological Nitrogen Removal in a Multiple Anoxic and Aerobic Biological Nutrient Removal Process

**DOI:** 10.1155/2015/531015

**Published:** 2015-09-30

**Authors:** Huoqing Wang, Yuntao Guan, Li Li, Guangxue Wu

**Affiliations:** Key Laboratory of Microorganism Application and Risk Control (MARC) of Shenzhen, Graduate School at Shenzhen, Tsinghua University, Shenzhen, Guangdong 518055, China

## Abstract

Two sequencing batch reactors, one with the conventional anoxic and aerobic (AO) process and the other with the multiple AO process, were operated to examine characteristics of biological nitrogen removal, especially of the multiple AO process. The long-term operation showed that the total nitrogen removal percentage of the multiple AO reactor was 38.7% higher than that of the AO reactor. In the multiple AO reactor, at the initial SBR cycle stage, due to the occurrence of simultaneous nitrification and denitrification, no nitrite and/or nitrate were accumulated. In the multiple AO reactor, activities of nitrite oxidizing bacteria were inhibited due to the multiple AO operating mode applied, resulting in the partial nitrification. Denitrifiers in the multiple AO reactor mainly utilized internal organic carbon for denitrification, and their activities were lower than those of denitrifiers in the AO reactor utilizing external organic carbon.

## 1. Introduction

Nitrogen and phosphorus in discharged wastewater can be key inducers for the eutrophication of receiving water bodies. As a protective environmental strategy, stringent nitrogen and phosphorus discharge standards from wastewater have been set in many countries, such as concentrations below 3 mg/L for total nitrogen (TN) and below 0.1 mg/L for total phosphorus (TP) in some USA areas [1]. Consequently, it is necessary to develop new or optimize the existing wastewater treatment technologies for compliance with the latest discharge standards.

Biological nitrogen removal is achieved by sequential nitrification under aerobic conditions and denitrification under anoxic conditions. During nitrification, ammonium is oxidized to nitrite by ammonium oxidizing bacteria (AOB) and then to nitrate by nitrite oxidizing bacteria (NOB). During denitrification, nitrite and/or nitrate is denitrified to nitrogen gas with organic carbon as the electron donor. Usually, predenitrification is widely applied for biological nitrogen removal, where denitrification occurs firstly in the anoxic phase by recycling nitrified wastewater from the following aerobic phase. In this type of anoxic and aerobic (AO) process, removal percentage of TN depends on the recycling ratio and organic carbon supplied. In order to achieve a high TN removal percentage, the recycling ratio should be increased and adequate organic carbon should be provided for complete denitrification. However, a high recycling ratio will bring the dissolved oxygen (DO) from the aerobic phase to the anoxic phase, reducing the organic carbon available for denitrification. In addition, in the conventional AO process, competition between denitrifiers and polyphosphate accumulating organisms (PAOs) also occurs and it is very difficult to achieve high removal efficiencies for TN and TP simultaneously [2]. Sometimes, postdenitrification is also adopted through endogenous respiration of heterotrophs for denitrification of the oxidized nitrogen, but the reaction rate of this process is relatively slow and requires long reaction duration. Therefore, postdenitrification by the addition of external organic carbons to enhance denitrification can be applied to enhance biological nitrogen removal, but this proves impeditive to the operational cost of wastewater treatment plants.

The multiple AO process is developed by optimizing the AO process, where intermittent aeration is adopted to achieve multiple nitrification and denitrification stages within one reaction phase in sequencing batch reactors (SBRs) or within one reaction zone in constant flow reactors. By this means, several advantages for improving biological nitrogen removal could be realized. Firstly, recycling mixed liquor between aerobic and anoxic phases will be reduced or omitted, resulting in a low concentration of DO recycled back to the anoxic phase and a low energy cost for recycling mixed liquor. Secondly, the competition between denitrifiers and PAOs is also alleviated, which can enhance phosphorus release during the anaerobic phase. Finally, alkalinity increases during denitrification in the anoxic phase can compensate for its reduction during nitrification in the aerobic phase, which can stabilize pH in the system and maintain high activities for both nitrifiers and denitrifiers [3, 4].

In the multiple AO process, when switching from the anoxic phase to the aerobic phase, nitrite accumulation often occurs due to the longer lag time of NOB than that of AOB, which may enhance shortcut nitrification and denitrification [5, 6]. By this means, not only nitrogen removal is improved, but also the aeration and organic carbon requirement is reduced [7–10]. In the multiple AO process, DO is an important factor affecting nitrification and denitrification efficiencies. Ruiz et al. [11] and Chuang et al. [12] found that, under low DO concentrations of 0.2–0.7 mg/L, partial nitrification could be easily achieved. While Ciudad et al. [13] found that, even under a DO concentration of 1.4 mg/L in activated sludge systems, nitrite accumulation still occurred, while in biofilm systems, Oyanedel-Craver et al. [14] found that nitrite accumulation even occurred with DO as high as 3.5 mg/L. Li et al. [2] found that, by using the intermittently operating mode, nitrite accumulation with a ratio to total oxidized nitrogen of above 64% could be achieved for DO in the range of 0.7–6 mg/L. From previous studies, different floc sizes and biofilm thicknesses might induce different microenvironmental conditions for the different nitrite accumulation phenomena [15]. Until now, characteristics of biological nitrogen removal in the multiple AO process are still not clear and further researches are required.

In this study, two SBRs, one with the conventional AO operating mode and the other with the multiple AO operating mode, were operated in the lab to examine characteristics of biological nitrogen removal. The conditions examined included long-term system operation, typical SBR cycles, and batch experiments for the examination of activities of both nitrification and denitrification. The research outputs would provide some new knowledge for advancing the multiple AO technology for enhanced biological nitrogen removal.

## 2. Materials and Methods

### 2.1. System Operation

Two parallel lab-scale SBRs, one of the common AO process and the other of the multiple AO process, were operated at 25°C. The working volume of the SBRs was 8 L. Each 24-hour period included 4 reaction cycles, and each cycle was 6 hours. The operation cycle of the AO SBR was 120 min anaerobic phase (including 10 min filling), 180 min aerobic phase, and settlement and withdrawal of treated wastewater of 60 min. The operation cycle of the multiple AO SBR was 120 min anaerobic phase (including 10 min filling), 30 min aerobic phase, 30 min anoxic phase, 30 min aerobic phase, 30 min anoxic phase, 60 min aerobic phase, and settlement and withdrawal of treated wastewater of 60 min. According to the study of Li et al. [2], the alternative anoxic phase of 30 min and aerobic phase of 30 min were able to inhibit the activities of NOB. The operations of the SBRs were controlled by timers for filling, mixing, aeration, and withdrawal. During aerobic phases, aeration was achieved through air pumps with micropore stones and the temperature was controlled by a heater. During anoxic or anaerobic phases, aeration was stopped and the reactors were stirred only by magnetic stirrers.

Each cycle, 4 litres of treated wastewater (3.5 L during the sludge discharging cycle) was discharged and 4 litres of influent wastewater was pumped into the reactors by peristaltic pumps. For each 24-hour period, during the final aerobic phase just before the settlement, 0.5 litres of mixed liquor was removed from both reactors to control the sludge retention time of around 16 days. The AO reactor was seeded with activated sludge taken from Nanshan Wastewater Treatment Plant, Shenzhen, China, while the multiple AO reactor was seeded with activated sludge taken from the AO reactor after more than three months of operation of the AO SBR.

The influent wastewater was made from the following components: 510 mg/L sodium acetate (NaAc), 153 mg/L NH_4_Cl, 14 mg/L CaCl_2_·2H_2_O, 90 mg/L MgSO_4_·7H_2_O, 46 mg/L Na_2_HPO_4_, 10 mg/L yeast extract, 200 mg/L NaHCO_3_, and 0.4 mL/L trace elements. The components of the trace elements were added according to Smolders et al. [16]. The influent wastewater contained the chemical oxygen demand (COD) concentration of around 400 mg/L, the ammonium nitrogen (NH_4_-N) concentration of around 40 mg/L, and the orthophosphate (PO_4_-P) concentration of around 10 mg/L.

### 2.2. Batch Experiments

For activated sludge under steady state, batch experiments were carried out to examine activities of nitrification and denitrification for activated sludge acclimated in the AO reactor and the multiple AO reactor. Average results of replications for each experiment were presented.

Batch nitrification experiments were carried out as follows. (1) Activated sludge was taken from the AO reactor and the multiple AO reactor just before the end of the last aerobic phase and centrifuged at 12000 rpm for 2 min, and then the supernatant was discarded. (2) The activated sludge was resuspended using the synthetic wastewater but without the addition of ammonium and acetate, and samples were taken for suspended solids (SS) and volatile suspended solids (VSS) measurement. (3) Ammonium was added to the mixed liquor from each reactor with the initial NH_4_-N concentration of 30 mg/L. (4) Batch nitrification experiments were started by aeration, and samples were taken at intervals of 10–15 min. NH_4_-N, nitrite nitrogen (NO_2_-N), and nitrate nitrogen (NO_3_-N) were measured and nitrification activities were obtained by linear regression of these parameters with time.

For batch denitrification experiments with the external organic carbon as the electron donor, after taking the activated sludge from the two SBRs before the end of the aerobic phase, acetate and nitrate were added to the mixed liquor with an initial concentration of 500 mg/L for sodium acetate and 30 mg/L for NO_3_-N. The batch reactors with the mixed liquor were sealed and mixed using magnetic stirrers, and the experiment was started. Samples were taken at intervals of 10–15 min and parameters of NO_2_-N, NO_3_-N, PO_4_-P, acetate, and polyhydroxybutyrate (PHB) were measured.

For batch denitrification experiments with the internal organic carbon as the electron donor, after taking the activated sludge from the two SBRs just before the end of the anaerobic phase, nitrite and nitrate were added to the mixed liquor with an initial concentration of 10 mg/L for NO_2_-N and 30 mg/L for NO_3_-N. The batch reactors with the mixed liquor were sealed and mixed using magnetic stirrers, and the experiment was started. Samples were taken at intervals of 10–15 min and parameters of NO_2_-N, NO_3_-N, and PHB were measured.

For batch denitrification experiments under organic carbon limited conditions, after taking the activated sludge from the two SBRs before the end of the aerobic phase (without external easily biodegradable organic carbon and limited internal organic carbon), ammonium and nitrite were added to the mixed liquor with an initial concentration of 10 mg/L for NH_4_-N of and 10 mg/L for NO_2_-N. The batch reactors with the mixed liquor were sealed and mixed using magnetic stirrers, and the experiment was started. Samples were taken at 10 min intervals and parameters of NH_4_-N, NO_2_-N, and NO_3_-N were measured.

### 2.3. Analytical Methods

COD, TN, TP, NH_4_-N, NO_3_-N, NO_2_-N, PO_4_-P, SS, and VSS were tested according to standard methods for the examination of water and wastewater [17]. pH and DO were measured using WTW portable pH meter (pH 3110, WTW, Germany) and DO meter (Oxi 315i, WTW, Germany), respectively.

PHB was tested according to the method of Karr et al. [18] and Rodgers and Wu [19]. Mixed liquor of 2 mL was taken from the SBRs and centrifuged at 12000 rpm for 2 min and the supernatant was discarded. The sludge was sequentially dewatered by 50%, 80%, and 96% ethanol solutions, each for 3 min, and then centrifuged at 12000 rpm for 2 min. After dewatering, the sludge was transferred to a glass tube with concentrated sulfuric acid twice, each time with a volume of 0.5 mL. The mixed liquor with concentrated sulfuric acid was digested at 100°C for 30 min and shaken every 10 min to mix the sample thoroughly. After digestion, 4 mL of deionized water was added to the glass tube, mixed, cooled to room temperature, and centrifuged at 12000 rpm for 2 min. The supernatant was then ready for testing of PHB with the HPLC (Shimadzu LC-20A, Japan). The HPLC had a UV detector at 210 nm and an Aminex HPLC Organic Acid Analysis Column (HPX-87H, Bio-Rad, USA). The mobile phase was 1‰ sulfuric acid with the flow rate of 0.6 mL/min. Acetate was also tested by HPLC with the procedure as those of the PHB testing.

## 3. Results and Discussion

### 3.1. Long-Term System Operation

After more than three months of operation, the system performance under steady state is shown in Table 1.

The average SS concentration was 3.16 g/L in the AO reactor and 3.68 g/L in the multiple AO reactor, with the VSS/SS ratio of 83% in both reactors. The effluent SS was 5.2 mg/L in the AO reactor and 9.0 mg/L in the multiple AO reactor. The measured synthetic wastewater contained concentrations for COD of 374 mg/L, NH_4_-N of 38.3 mg/L, and PO_4_-P of 9.8 mg/L. Both reactors removed COD, NH_4_-N, TN, and TP efficiently, with their removal percentages of 90.8%, 99.8%, 67.9%, and 95.7% in the AO reactor and 88.4%, 100%, 94.2%, and 92.2% in the multiple AO reactor. For the filtered and unfiltered effluent, there was not much difference in COD, TN, and TP concentrations due to the low effluent SS concentration (below 10 mg/L) in both reactors.

### 3.2. Typical SBR Cycle Performance

**Figure 1:**
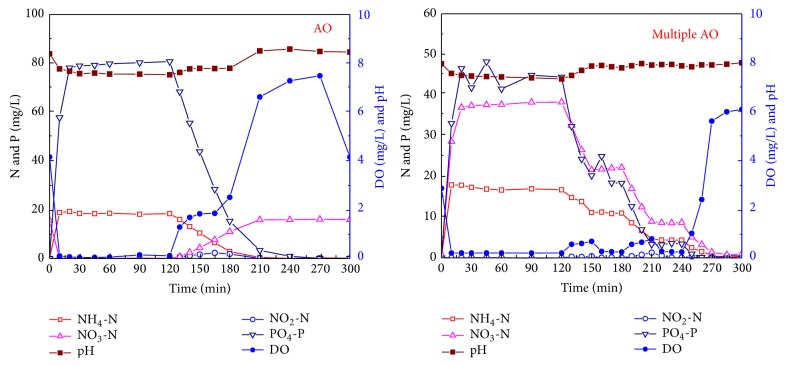
Dynamics of various parameters during typical cycles in the AO and multiple AO reactors.

For a typical SBR cycle, the dynamics of nitrogen, phosphorus, pH, and DO in the two reactors are shown in Figure 1.

In the AO reactor, during a typical cycle, ammonium increased during the fill step and peaked at the end of the fill step; its concentration remained stable during the anaerobic phase and then nitrified to nitrite and nitrate during the aerobic phase. Nitrite was accumulated during the initial aerobic phase and then decreased with further nitrification. Nitrate denitrified quickly during the fill step and then produced during nitrification in the aerobic phase with the concentration reaching around 15.9 mg/L. During the anaerobic phase, PAOs released the phosphorus and the phosphorus concentration reached to 86.4 mg/L after 2 h anaerobic reaction. During the aerobic phase, PO_4_-P was taken up with a final concentration of less than 1.0 mg/L.

In the multiple AO reactor, during the typical cycle, similar phenomena were observed as those in the AO reactor. During the aerobic phase, NO_2_-N was accumulated during the initial stage of the final aerobic phase with the maximum concentration reaching 1.6 mg/L. There was no obvious NO_3_-N produced during the alternative anoxic and aerobic phases, while it increased during the final 1 hour aerobic phase and reached to 1.4 mg/L. For PAOs, PO_4_-P was released during the anaerobic phase and then taken up during the aerobic phase, while it did not vary much during the anoxic phase.

In both reactors, the pH remained in the range of 7.5–8.5, which was benefit for the activities of nitrifiers or PAOs. During the aerobic phase, due to activities of nitrifiers and heterotrophs, DO increased slowly during the initial aerobic phase and reached to above 6 mg/L after 1 hour when ammonium was nitrified completely in the AO reactor. In the multiple AO reactor, DO concentrations remained low before the final aerobic phase, with concentrations below 1 mg/L, while during the final aerobic phase, DO concentration increased with the complete of nitrification and finally reached to around 5.7 mg/L.

### 3.3. Batch Nitrification Experiments

**Figure 2:**
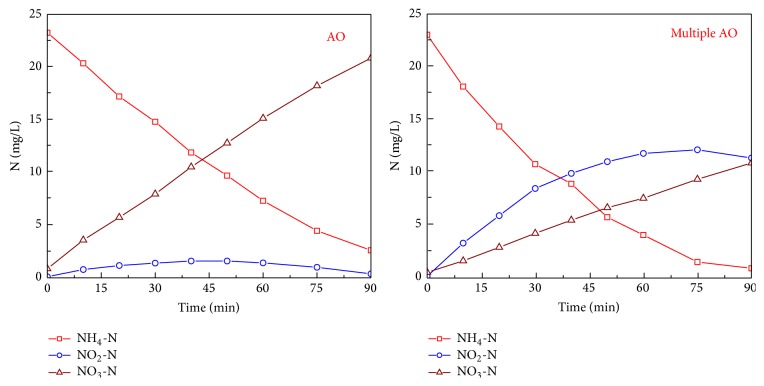
Dynamics of various types of nitrogen during the batch nitrification for activated sludge taken from both AO and multiple AO reactors.

The batch nitrification experiment results for activated sludge taken from both the AO reactor and the multiple AO reactor are shown in Figure 2.

For activated sludge taken from the AO reactor, with the reduction of ammonium, nitrate production occurred with only a small amount of nitrite accumulated. The NH_4_-N reduction rate was 6.6 mg N/g VSS/h and the NO_3_-N production rate was 5.6 mg N/g VSS/h; NO_2_-N accumulated during the initial 40 minutes, peaked at 1.6 mg/L, and then decreased to below 0.4 mg/L.

For activated sludge taken from the multiple AO reactor, the reduction rate of NH_4_-N was 8.1 mg N/g VSS/h, and its concentration was below 1 mg/L after 90 minutes reaction. With the reduction of ammonium, accumulation of nitrite was expected, and its accumulation rate was 5.5 mg N/g VSS/h during the initial 30 minutes. The NO_2_-N concentration peaked at 12.1 mg/L after 75 minutes reaction and then decreased thereafter. The NO_3_-N production rate was 2.3 mg N/g VSS/h over the whole reaction phase.

### 3.4. Batch Denitrification Experiments

**Figure 3:**
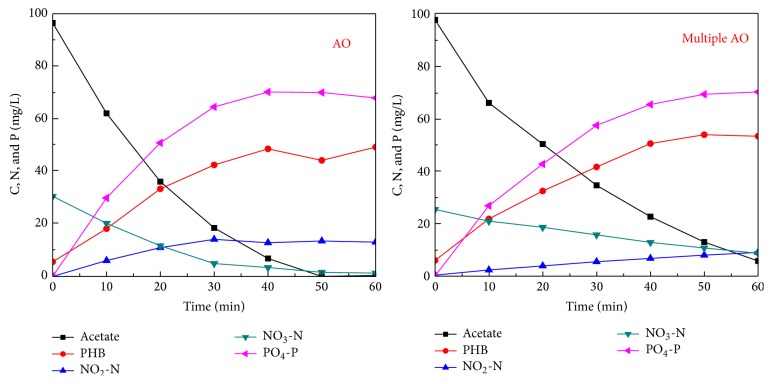
Dynamics of various types of nitrogen, organic carbon, and phosphorus during denitrification with external organic carbon as the electron donor for activated sludge taken from both AO and multiple AO reactors.

The results of batch denitrification experiments with the external organic carbon as the electron donor are shown in Figure 3. For activated sludge taken from the AO reactor, during the initial 30 minutes, with an adequate supply of acetate, the NO_3_-N reduction rate was 31.9 mg N/g VSS/h and then decreased thereafter; accompanied with the reduction of nitrate, NO_2_-N was accumulated with the accumulation rate of 17.7 mg N/g VSS/h during the initial 30 minutes, and the NO_2_-N concentration was stable during the final 30 minutes with the consumption of acetate. For activated sludge taken from the multiple AO reactor, denitrification was carried out slowly with the external organic carbon as the electron donor, with a NO_3_-N reduction rate of 10.2 mg N/g VSS/h and a nitrite accumulation rate of 5.3 mg N/g VSS/h. The acetate utilization rate was 96.9 mg C/g VSS/h in the AO reactor during the initial 30 minutes, and it was 54.6 mg C/g VSS/h in the multiple AO reactor over the whole reaction period. Acetate was partially stored as PHB and the PHB production rate was 46.9 mg/g VSS/h in the AO reactor during the initial 30 minutes and 40.6 mg/g VSS/h in the multiple AO reactor over the whole reaction period. During the initial 30 minutes, the PO_4_-P release rate was 80 mg P/g VSS/h in the AO reactor and 70.2 mg P/g VSS/h in the multiple AO reactor.

**Figure 4:**
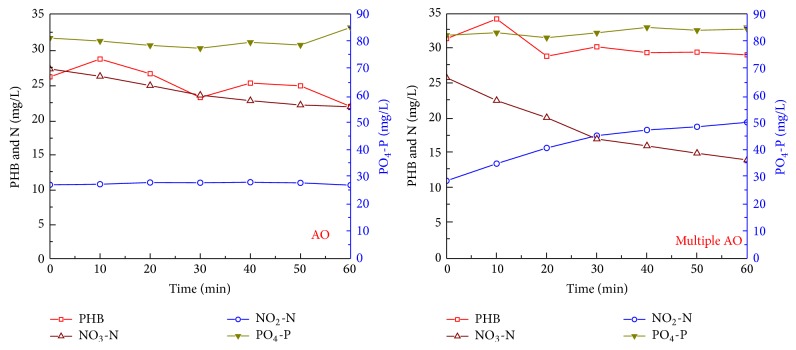
Dynamics of various types of nitrogen, PHB, and phosphorus during denitrification with internal organic carbon as the electron donor for activated sludge taken from both AO and multiple AO reactors.

The results of batch denitrification experiments with the internal organic carbon as the electron donor are shown in Figure 4. For activated sludge taken from the AO reactor, denitrification was slow, with the NO_3_-N reduction rate of 2.2 mg N/g VSS/h, and the NO_2_-N concentration remained relatively stable. For activated sludge taken from the multiple AO reactor, during the initial 30 minutes, the NO_3_-N reduction rate was 5.6 mg N/g VSS/h and the NO_2_-N production rate was 4.2 mg N/g VSS/h, while, during the latter 30 min, the NO_3_-N reduction rate was 1.9 mg N/g VSS/h and the NO_2_-N production rate was 1.2 mg N/g VSS/h.

**Figure 5:**
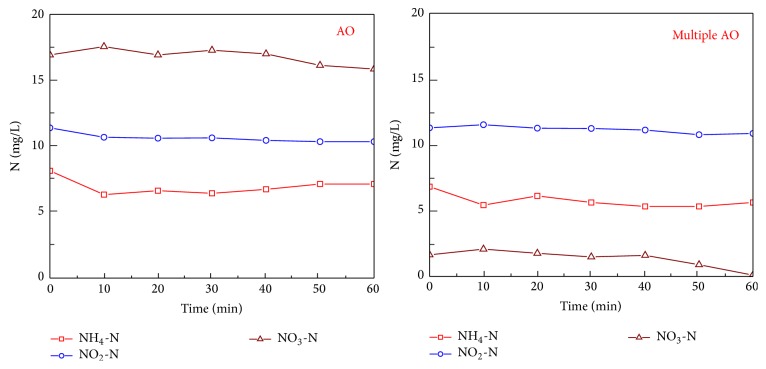
Dynamics of various types of nitrogen during denitrification under organic carbon limited conditions for activated sludge taken from both AO and multiple AO reactors.

The results of batch denitrification experiments during organic carbon limited conditions are shown in Figure 5. For activated sludge taken from the AO reactor, denitrification was very slow, and the NO_3_-N reduction rate was 0.89 mg N/g VSS/h and the NO_2_-N reduction rate was 0.4 mg N/g VSS/h. For activated sludge taken from the multiple AO reactor, the NO_3_-N reduction rate was 0.78 mg N/g VSS/h and the NO_2_-N reduction rate was 0.23 mg N/g VSS/h. Under all conditions, the concentration of ammonium was stable, indicating that no anaerobic ammonium oxidation occurred. The reduced oxidized nitrogen was mainly due to endogenous respiration.

## 4. Discussion

In the multiple AO technology, by adopting the multiple alternative AO process, simultaneous nitrification and denitrification could be enhanced and complete nitrification would occur in the final aerobic phase. In the present study, the multiple AO reactor shortened the aerobic phase by 33.3% compared with the AO reactor. Under steady state, the multiple AO reactor improved the TN removal percentage by 38.7% compared with the AO reactor. Therefore, the multiple AO process could improve TN removal significantly. Similar results were also obtained for TN removal from municipal wastewater and slaughterhouse wastewater by using the multiple AO processes [2, 20]. Li et al. [2] obtained a TN removal percentage of 96% in an intermittently aerated SBR for treating high ammonium concentration slaughterhouse wastewater. Sasaki et al. [20] obtained a removal percentage of TN of 92% by using two-stage intermittently aerated reactors treating municipal wastewater.

During the initial SBR cycle, with the reduction of NH_4_-N, no accumulation of both NO_2_-N and NO_3_-N was observed in the multiple AO reactor, indicating simultaneous nitrification and denitrification occurred inside the reactor [7]. By adopting an alternative anoxic phase of 30 min and aerobic phase of 30 min, no NO_3_-N accumulation was observed during the initial 2 hours, and even during the first hour of the final aerobic phase, the NO_3_-N concentration remained at a very low concentration of below 0.15 mg/L during the initial 30 min, which finally increased to above 1.4 mg/L during the final stage. Li et al. [2] and Zhang et al. [6] observed in their intermittently aerated systems that activities for NOB had recovered after 60 min and 16–18 min after recovering from the anoxic conditions. Therefore, the operating mode of an alternative anoxic and aerobic mode could induce nitrite accumulation and result in shortcut nitrification and denitrification.

From the batch nitrification experiment, a higher NH_4_-N nitrification rate was observed in the multiple AO reactor than that in the AO reactor, while a low NO_2_-N nitrification rate was obtained from the multiple AO reactor. Therefore, a high nitrite accumulation potential and a peak NO_2_-N concentration of 12.1 mg/L were obtained from the multiple AO reactor. These results showed that the multiple AO operating mode could inhibit the activities of NOB. In the multiple AO reactor, DO was in the range of 0.4–0.7 mg/L during the initial anoxic and aerobic alternative stage, which might also inhibit activities of NOB and resulted in the shortcut nitrification and denitrification [11, 12]. Therefore, in the multiple AO reactor, due to the low DO concentration and the alternative anoxic and aerobic operating mode, NOB activities were inhibited, and once nitrified NH_4_-N to NO_2_-N, denitrifiers could use NO_2_-N directly for denitrification through the shortcut denitrification process [21, 22].

When the external organic carbon was used as the electron donor for denitrification, a high denitrification rate was obtained in the AO reactor, while when the internal organic carbon was used as the organic carbon, a high denitrification rate was obtained in the multiple AO reactor. The reason could be due to the fact that, in the multiple AO reactor, the organic carbon was mainly stored as the internal organic carbon and then utilized by both PAOs and denitrifiers. Therefore, the denitrification rate for denitrifiers from the multiple AO reactor was relatively low with the utilization of the internal organic carbon, because utilization of PHB usually limited the biological reactions as shown by some previous studies [23]. Zeng et al. [7] found that, in the anoxic/aerobic biological nitrogen and phosphorus removal systems, COD in the influent was mainly stored by PAOs or GAOs as polyhydroxyalkanoate (PHA) and then used later for phosphorus uptaken or denitrification. Mino et al. [24] and Smolders et al. [16] found that when acetate was used as the external organic carbon, it would be mainly stored as PHB. Similarly, in the present study, acetate would be also accumulated as PHB during the anaerobic phase and then used for denitrification during the alternative AO phases, which could improve the utilization efficiency of organic carbons and enhance biological nitrogen removal, such that a high TN removal percentage of 94.2% was obtained in this study.

In the multiple AO reactor, during the initial fill step, only a minor concentration of oxidized nitrogen existed inside the reactor and this also inhibited the acclimation of denitrifiers which could utilize the external organic carbon. This was also confirmed from the batch denitrification experiments. For activated sludge taken from the multiple AO reactor, the denitrifying rate only decreased from around 10.2 mg N/g VSS/h using external organic carbon to 5.6 mg N/g VSS/h using internal organic carbon, indicating that a high proportion of denitrifiers using internal organic carbons was acclimated in this reactor. While in the AO reactor, during the fill and anaerobic phase, denitrifiers which could utilize external organic carbon for denitrifying oxidized nitrogen remaining from the previous cycle were acclimated. This was also confirmed from the batch denitrification experiments. For activated sludge taken from the AO reactor, the denitrifying rate was decreased from around 31.9 mg N/g VSS/h using external organic carbon to 2.2 mg N/g VSS/h using internal organic carbon, indicating that a high proportion of denitrifiers using external organic carbons was acclimated in this reactor. Therefore, different types of denitrifiers might be acclimated in both reactors and this should be recognized when modelling denitrification with different operating modes.

## 5. Conclusions

(1) As to the influent COD of 374.4 mg/L, NH_4_-N of 38.3 mg/L, and PO_4_-P of 9.8 mg/L, the removal percentages of COD, NH_4_-N, TN, and TP were 90.8%, 99.8%, 67.9%, and 95.7% in the AO reactor and were 88.4%, 99.9%, 94.2%, and 92.2% in the multiple AO reactor. In the multiple AO reactor, the TN removal percentage increased by 38.7% by adopting alternative anoxic and aerobic phases. (2) In the multiple AO reactor, during the initial alternative anoxic and aerobic phase, due to simultaneous nitrification and denitrification, no obvious nitrite or nitrate accumulation was observed. (3) In the multiple AO reactor, due to the applied alternative anoxic and aerobic mode and low DO concentrations during the initial aerobic phase, NOB was inhibited and a low nitrite nitrification rate was obtained. (4) Denitrifiers in the multiple AO reactor mainly utilized internal organic carbon as the electron donor for denitrification and its denitrifying activities were lower than those in the AO reactor where denitrifiers mainly utilized external organic carbon.

## Figures and Tables

**Table 1:** Influent and effluent water quality from the AO and multiple AO reactors (unit of mg/L).

	Influent	Effluent from the AO reactor	Effluent from the multiple AO reactor

COD	374	32.9	39.4
NH_4_-N	38.3	0.06	0.02
NO_2_-N	—	0.02	0.11
NO_3_-N	—	12.3	1.2
TN	—	12.3	2.2
PO_4_-P	9.8	0.26	0.25
TP	—	0.42	0.76
